# Assessing the climate adaptive potential of imported Chili in comparison with local cultivars through germination performance analysis

**DOI:** 10.1186/s12870-024-05168-4

**Published:** 2024-06-14

**Authors:** Farhan Ahmad, Kusumiyati Kusumiyati, Mochamad Arief Soleh, Muhammad Rabnawaz Khan, Ristina Siti Sundari

**Affiliations:** 1 Department of Agronomy, Agricultural Faculty, Universitas Padjadjaran, Jl. Bandung- Sumedang km 21 Jatinangor, Sumedang, West Java, Indonesia; 2 Department of Agronomy, Faculty of Crop Production Sciences, The University of Agriculture Peshawar, Khyber Pakhtunkhwa, Peshawar Pakistan; 3 Department of Agribusiness, Faculty of Agriculture, Universitas Perjuangan, Jl. PETA No. 177, Tasikmalaya, West Java Indonesia

**Keywords:** Seed germination, Resilience, Climate adaptation, Agricultural diversity, Environmental factors

## Abstract

**Background:**

The study offers insightful information about the adaptability of local and imported Chili cultivars. This experiment examines how three different chili cultivars Tanjung, Unpad, and Osaka perform in the germination and early growth phases while considering a wide range of environmental conditions. Research conducted in Jatinangor, Sumedang Regency, Indonesia, highlights the differences between cultivars and the varied possibilities for adaptability each variation possesses.

**Results:**

Among them, Tanjung stands out as the most promising cultivar; its robust performance is demonstrated by its high germination index 91.7. Notable features of Osaka include the highest biomass output (1.429 g), the best water usage efficiency (WUE) at 0.015 g/liter, and the best distribution uniformity (91.2%) and application efficiency (73.6%) under different irrigation conditions. Tanjung’s competitiveness is further evidenced by the fact that it trails Osaka closely on several metrics. Lower performance across criteria for Unpad suggests possible issues with flexibility.

**Conclusion:**

The value of this information becomes apparent when it comes to well-informed breeding programs and cultivation techniques, especially considering uncertain climate patterns and global climate change. This research contributes significantly to the body of knowledge, enabling well-informed choices for environmentally dynamic, sustainable chili farming.

## Introduction

Chili is one of the most extensively grown and consumed vegetable crops worldwide (*Capsicum annuum* L.) [1]. Because of its high vitamin, antioxidant, and capsaicinoid content, it has medicinal benefits and great nutritional and economic worth. Chili is commonly grown as a spice, vegetable, or cash crop in the tropics [2]. 7.18 million tons of chili are produced worldwide on 1.5 million hectares [3]. Indonesia is one of the world’s top producers of chili peppers, making chili growing a significant agricultural sector in the nation. Indonesia produces about 5% of the world’s total chilies yearly. With a total output of 2.7 million tons, Indonesia was the third-largest producer of chilies worldwide in 2019 [4]. One vegetable commodity that offers excellent and potential business opportunities in Indonesia is chili. The demand for chili rises annually in tandem with the country’s growing population and the emergence of sectors that use chili as a raw material [5].

The unpredictable local climates and climate change present significant challenges for modern agriculture [6]. The resilience and sustainability of agriculture are significantly impacted by the ability of chili cultivars to adapt to various environmental conditions [7]. Variable climate regimes can be sustained by cultivars with robust adaptation features, which ensures farmers stable yields and economic viability [8, 9]. It is essential to comprehend the responses of various chili cultivars to diverse environmental situations to design resilient and adaptable crops [10]. Unpredictable results might arise from introducing exotic chili cultivars into a new environment, impacting genetic diversity and crop performance [11]. Chili peppers are widely cultivated in various soil types and climates, and they play a significant role in global agriculture and culinary traditions [12]. Because of the globalization of the food trade, there has been a worldwide interaction of chili cultivar, exposing exotic cultivars to new habitats and posing problems regarding their ability to adapt compared to locally adapted genotypes [8].

Chili peppers’ germination, the first stage of their life cycle, is crucial to crop establishment and total yield success [13]. It symbolizes the vital change from a dormant seed to a seedling that is actively growing, made possible by the uptake of water and the start of metabolic activities [14]. Choosing and breeding chili varieties with improved adaptation features is critical because climate change is putting unprecedented strain on agricultural systems [15]. Comprehending the germination patterns of diverse chili genotypes can offer valuable information about their capacity to adjust to various environmental factors, including light intensity, moisture content, humidity, wind speed, and temperature [16]. One of the most critical aspects of the plant life cycle, germination, is what determines crop establishment success and total yield [17]. Chili peppers are exposed to a wide range of environmental conditions, evaluating their adaptability requires a grasp of the nuances of their germination performance [18]. This scenario is further complicated by genetic variety within chili populations when imported varieties introduce new genetic material into local gene pools [19].

It is essential to comprehend how imported chili cultivars perform relative to native types in Indonesia, where chili growing is essential to the agricultural industry [20]. Diversifying genetic resources and improving responsiveness to local environmental circumstances arise with the introduction of imported cultivars [21]. Measuring how these imported cultivars’ germination performs compared to native provides information about how effectively they integrate into Indonesian agroecosystems, which can lead to improvements in the region’s methods of producing chilies [22].

## Importance of introduction cultivar

Tanjung and Unpad, two native cultivars, have moderate to high productivity levels. They normally yield between 5.3 and 18.5 tons per hectare and 14.6 to 23.6 tons per hectare, respectively. On the other hand, compared to native cultivars, the imported variety Osaka of chilies exhibits a remarkable production potential of almost 30 tons per hectare, indicating significantly better yields. Moreover, Osaka’s disease resilience offers an additional enticement, implying improved robustness and reliability in agricultural practices. Osaka’s high yield potential and disease resilience highlight its applicability in farming environments where risk mitigation and production maximization are critical factors.

By thoroughly comparing the germination performance of imported chili varieties to indigenous genotypes, this study seeks to uncover the adaptive potential of these cultivars. The results of this study not only advance our knowledge of the biology of chili peppers but also have more significant implications for crop management, sustainable agriculture, and food security, given the threat of climate change.

## Materials and methods

### Research locality and materials

The research was conducted at the Bale Tatanen nursery house, Faculty of Agriculture, Padjadjaran University, Jatinangor, Sumedang Regency, at an altitude of 685 m above sea level and Field Laboratory from October 2023 – December 2023. The materials that were used in the experiments comprising plastic container, laboratorium, knife, stick, scales, wrap, packaging, box, thermometer, hydrometer, thermohygrometer, stationary, label, scale glass, gallon 200 l, lux meter, anemometer, calliper, chlorophyl meter (Konica Minolta SPAD-502Plus (Tokyo, Japan). Three different cultivars were investigated in the climate adaptation experiment that was carried out at Jatinangor, Sumedang City. Among these were two locally sourced cultivars, UNPAD and Tanjung, and the Osaka cultivar, which was imported from Japan. The assessment parameters comprised both performance indicators and the capacity to adjust to the particular subtleties of the surrounding environment.

### Processing of seeds

Each cultivar’s seeds were tested for viability by immersing them in hot water having a temperature of 50–58 for 30 min before being put in the germination trays. Before beginning the growing procedure in the trays, this pre-germination step acted as a test to ascertain the likelihood of successful germination. Germination trays with drainage holes were used to avoid waterlogging. To reduce the chance of infection, the trays were thoroughly cleaned and sanitized. Half the trays were filled with cocopeat and charcoal (Because of its superior aeration, near-neutral pH, and capacity to retain water, cocopeat is utilized in germination trays to facilitate the healthy growth of seedlings. By enhancing drainage, absorbing toxins, and possessing natural antifungal and antibacterial qualities, charcoal shields seedlings from diseases. They work in tandem to produce the ideal growth environment for adequate germination) in a 2:1 ratio. This medium gave seedlings the nutrients they needed and was well-draining. A hard but not compacted surface was produced after leveling and lightly pressing the media surface in the tray. Thus, optimal seed to media contact is ensured. Maintaining a planting depth that is advised for each kind of seed (Every seed was kept between 0.5 and 1 centimetres deep. This depth assures a light layer of soil or growing media over the seeds, which promotes ideal germination conditions and simple seedling emergence). Using a little shovel, indentations or furrows were made in the media. After planting the seeds at the proper distance apart, a thin layer of media was applied over them. A watering spray bottle was used to water the seeds lightly. Avoiding overwatering was advised since it could cause damping off and other fungal disease. The nursery house with indirect sunlight was where the germination trays were kept (Indirect sunlight protects young seedlings from the extreme heat and light that can lead to dehydration and stress in the nursery house. It assures them enough light for photosynthesis without the risk of drying out or burning). Ensure enough light for the seeds to germinate and the seedlings to flourish. Every media was consistently tested for moisture content, (Moisture levels were checked twice daily (Morning and evening) before irrigation. Adjusting environmental conditions means placing the germination trays out of direct sunlight to avoid heat stress) the cover was taken off after the seedlings appeared, and environmental conditions were changed to suit the needs of the plants.


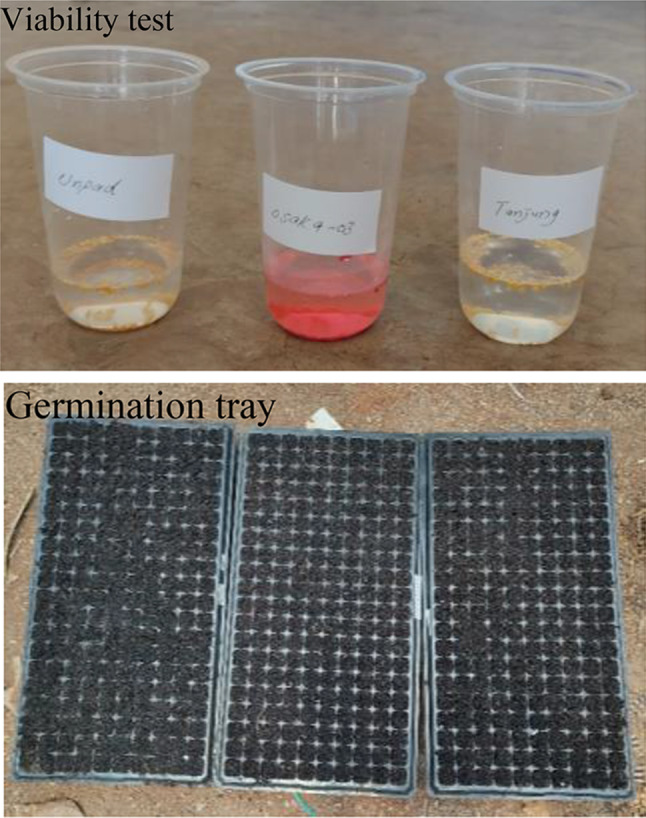


### Data collection

#### Germination index

$$GI\, = \,\frac{{Germinated \, seeds }}{{Days\,to\,first\,count}}\, + \, \ldots \, + \,\frac{{Germin ated\,seeds}}{{Days\,to\,final\,count}}$$

### Number of lateral roots

$$\begin{aligned} Average\, & No.\,of\,lateral\,roots \\ & = \,\frac{{Total\,number\,of\,lateral\,roots\,for\,a\,cultivar }}{{Number\,of\,seedlings\,for\,that\,cultivar }} \\ \end{aligned}$$

### Root-shoot length ratio

$$\begin{aligned} Root{\mkern 1mu} & - {\mkern 1mu} shoot{\mkern 1mu} \, length{\mkern 1mu} \, ratio \\ & = {\mkern 1mu} \frac{{Total{\mkern 1mu}\, root{\mkern 1mu} \, length}}{{Total{\mkern 1mu} \, shoot{\mkern 1mu}\, length}} \\ \end{aligned}$$

#### Vigor index

$$\begin{aligned} Vigor{\mkern 1mu} & \, index{\mkern 1mu} \\ & = {\mkern 1mu} \left( {\frac{{Germin ation{\mkern 1mu}\, rate{\mkern 1mu} \times {\mkern 1mu} Seedling{\mkern 1mu}\, length{\mkern 1mu} \times {\mkern 1mu} Root{\mkern 1mu}\, length}}{{100}}} \right) \\ \end{aligned}$$

### Water absorption capacity (g/seed)

$$\begin{aligned} Water\, & absorption\,capacity\,\left( {\frac{g}{{seed}}} \right) \\ & = \,{\text{Final}}\,{\text{weight}}\,{-}\,{\text{Initial}}\,{\text{dry}}\,{\text{weight}} \\ \end{aligned}$$

### Water absorption index (Seeds)

$$\begin{aligned} Water\, & absorption\,index \\ & = \,\left( {\frac{{Final\,weight\, - \,Initial\,dry\,weight}}{{Initial\,dry\,weight}}} \right)\, \times \,100 \\ \end{aligned}$$

### Irrigation efficiency observation

#### **Water use efficiency (g/liter)**

Computed as the biomass produced divided by the water applied.

$$\begin{aligned} Water\, & use\,efficiancy\,\left( {\frac{g}{{liter}}} \right) \\ & = \,\left( {\frac{{Total\,biomass\,of\,seedling\left( g \right)}}{{Water\,applied}}} \right) \\ \end{aligned}$$

### Application efficiency (%)

The portion of water provided that goes toward retaining soil moisture.

$$\begin{aligned} Application\, & efficiancy\,\left( \% \right) \\ & = \,\frac{{Water\,hold\,by\,plant\,media\left( {root\,zone} \right)}}{{Water\,applied}}\, \times \,100 \\ \end{aligned}$$

### **Distribution uniformity (%)**

Demonstrates how evenly water has been applied throughout the research area.

$$\begin{aligned} Distribution\, & uniformity{\mkern 1mu} \left( \% \right) \\ & = {\mkern 1mu} \frac{{Water{\mkern 1mu}\, applied{\mkern 1mu}\, to{\mkern 1mu} \, quarter{\mkern 1mu}\, of{\mkern 1mu} \, area{\mkern 1mu} }}{{Average{\mkern 1mu} \, depth{\mkern 1mu} \, of{\mkern 1mu} \, water{\mkern 1mu}\, applied}}{\mkern 1mu} \times {\mkern 1mu} 100 \\ \end{aligned}$$

### **Irrigation efficiency (%)**

The overall effectiveness of plant water utilization.

$$\begin{aligned} Irrigation\, & efficiency\,\left( \% \right) \\ & = \,\frac{{Beneficial\,water\,use}}{{Total\,water\,applied}}\, \times \,100 \\ \end{aligned}$$

### Climatic parameters

**Fig. 1:**
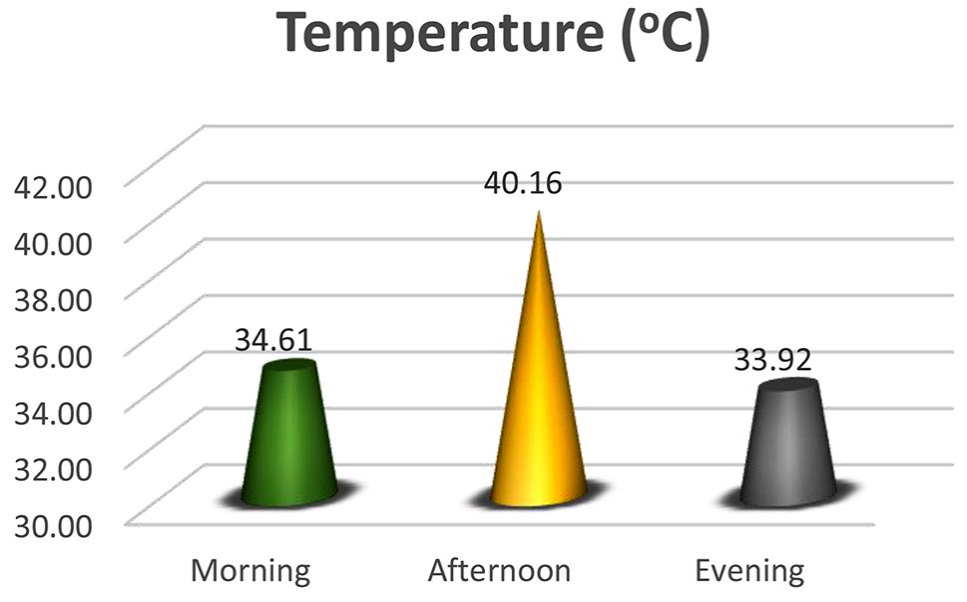
Temperature (^o^C) was recorded in the morning, afternoon, and evening at the experimental site

**Fig. 2:**
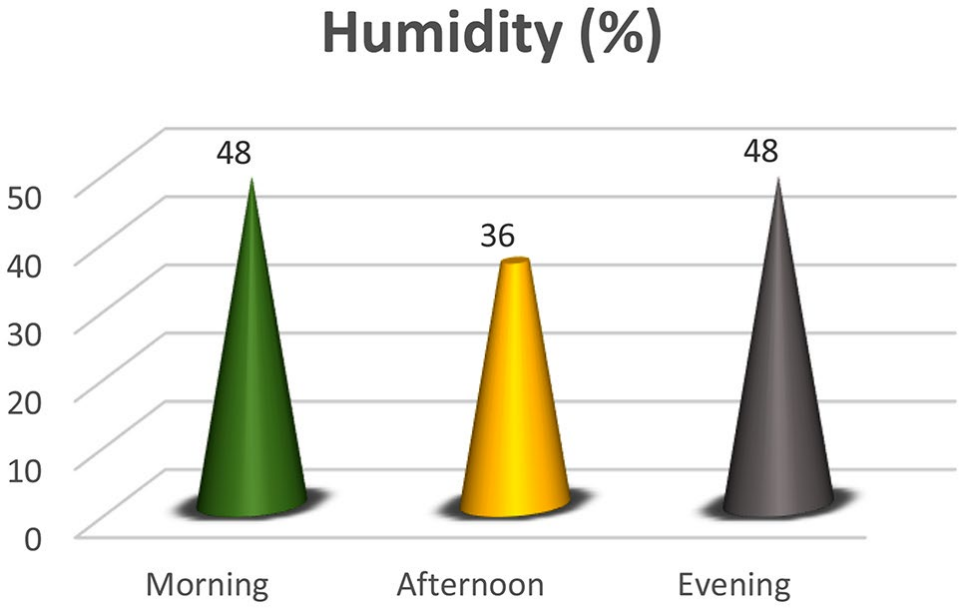
Humidity (%) was recorded in the morning, afternoon, and evening at the experimental site

**Fig. 3:**
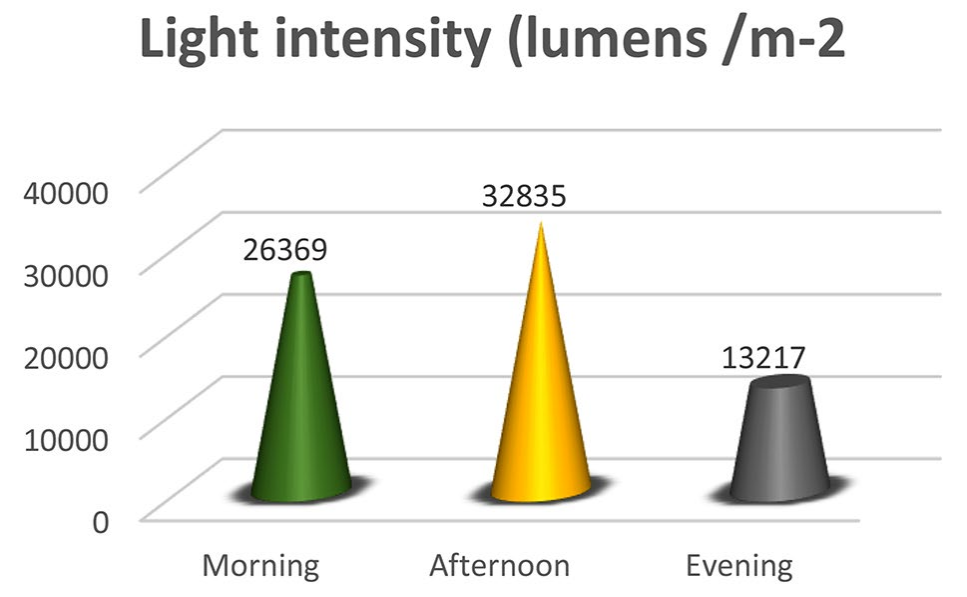
Light intensity (lumens/m-2) was recorded in the morning, afternoon, and evening at the experimental site

At the study site-nursery house, temperature, humidity, and light intensity measurements were taken systematically over every phase of the investigation. Data were collected three times daily in the morning, afternoon, and evening to grasp the daily fluctuations thoroughly. The experimental space was equipped with strategically placed digital thermometers for recording temperature (Fig. 1), hygrometers for humidity (Fig. 2), and lux meters for light intensity (Fig. 3) to ensure representative sampling at various times of the day. The data were recording daily at morning (6–8 am), afternoon (11 − 1 pm) and evening (4–6 pm) daily.

### Statistical analysis

Four replications involving 50 seeds were used in the completely randomized factorial design of the treatments. ANOVA with several factors was applied to the data. If the F test was significant at *p* ≤ 0.05, the mean separation was carried out using the Tukey test (LSD 0.05) (*).

The ANOVA model for a completely randomized factorial design with two factors (A and B) and their interaction (AB) is represented conceptually as follows:

$$Yijk = \hspace{0.17em}+\hspace{0.17em}I +j +\left( {{{\alpha \beta }}} \right)\,ij +ijk$$

### Tukey test for mean separation

Further analysis was conducted to determine specific differences between treatment means for a significant difference (*p* ≤ 0.05). The Tukey HSD test is a post-hoc test frequently used for mean separation in ANOVA; in this study, it is represented by LSD 0.05.

The following is the formula to determine Tukey’s HSD:

$$HSD\, = \,q\, \times \,\sqrt {MS\,{{error} \mathord{\left/{\vphantom {{error} n}} \right.\kern-\nulldelimiterspace} n}}$$

Where n is the amount of data per group, MS error is the mean square error from the ANOVA, and q is the crucial value from the studentized range distribution.

### Brief overview of methodology

This study used an array of essential steps in its methodology for the germination performance analysis. Using hot water immersion, the viability of seeds from three cultivars of chilies Tanjung, Unpad, and Osaka was first assessed. Then, germination trays filled with well-draining media were sowed at the proper depths and spacing. The trays were placed in a nursery house under indirect sunlight to promote germination. Environmental factors and media moisture levels were observed during the germination phase. The observations were assessed to evaluate each cultivar’s performance after germination. A statistical analysis was performed to determine how effectively different chili cultivars’ germination performed. This thorough research yielded important information for sustainable agricultural operations by illuminating the cultivars’ resilience and adaptability to various environmental circumstances.

## Results and discussion

**Table 1:** Data showing the significancy (*P* < 0.05) of each recorded observation

Observations		Germination index	Lateral roots	Root-shoot length ratio	Vigor index	Water absorption capacity (g/seed)	Water absorption index (%)	Leaf chlorophyll index	Seedling biomass (g)
**Cultivars**	*C1-Tanjung*	0.002	0.012	0.032	0.000	0.011	0.005	0.035	0.010
	*C2-Unpad*								
	*C3-Osaka*								

Note The mean values less than (*P* < 0.05) indicate the significant differences among cultivars

**Table 2:** Mean values (mean ± SE) of the recorded observations

Observations		Germination index	Lateral roots	Root-shoot length ratio	Vigor index	Water absorption capacity (g/seed)	Water absorption index (%)	Leaf chlorophyll index	Seedling biomass (g)
Cultivars	*C1 - Tanjung*	91.7 ± 0.84	3.14 ± 0.02	0.416 ± 0.01	495.04 ± 7.48	0.026 ± 0.005	1.370 ± 0.02	35.14 ± 0.74	1.068 ± 0.15
	*C2- Unpad*	57 ± 1.17	2.47 ± 0.02	0.529 ± 0.01	250.20 ± 7.76	0.028 ± 0.003	1.422 ± 0.07	34.80 ± 0.91	0.857 ± 0.06
	*C3- Osaka*	84 ± 0.93	4.7 ± 0.10	0.731 ± 0.01	538.40 ± 2.24	0.024 ± 0.002	1.260 ± 0.07	37.10 ± 0.72	1.430 ± 0.15

**Table 3:**
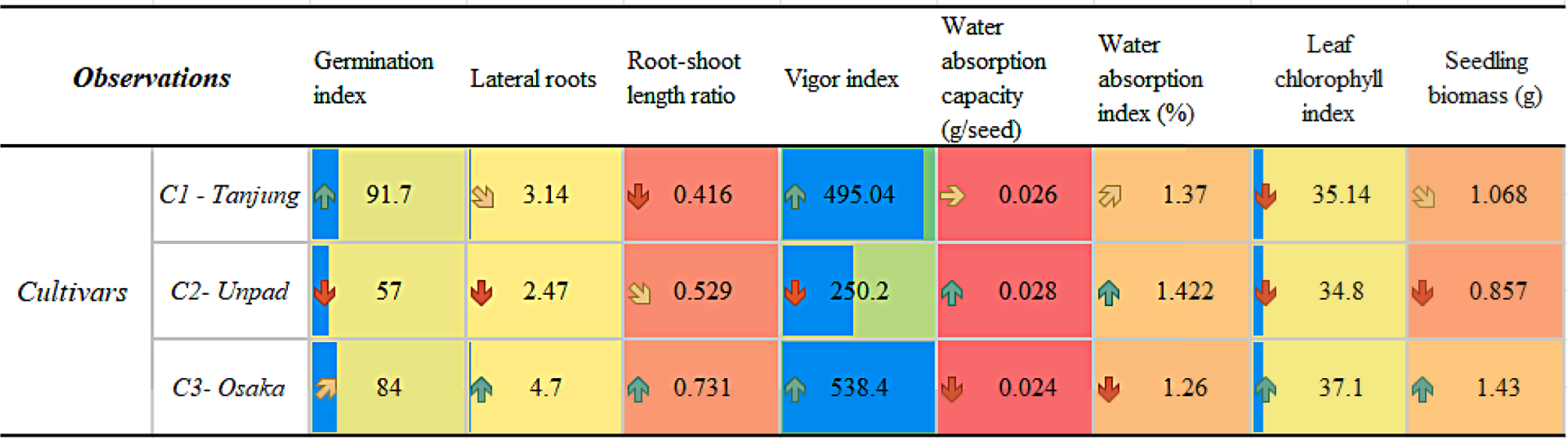
Heat map of the observed parameter mean values

### Germination index

**Fig. 4:**
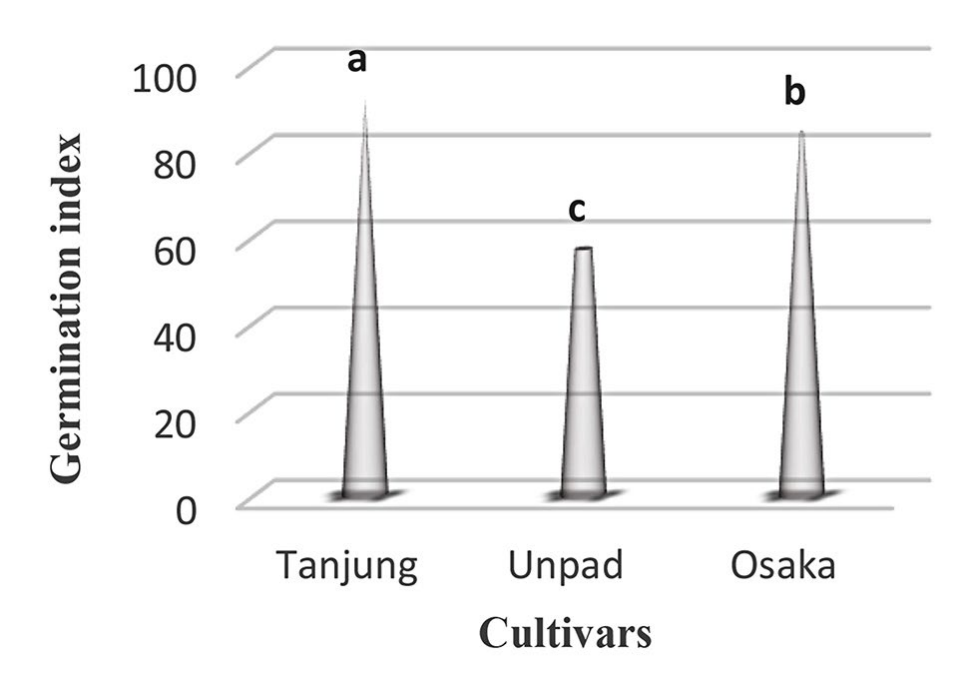
Mean germination index in cultivars studied under the climatic parameters. (The letters a, b and c indicating the significant differences in cultivars)

A statistically significant (*p* < 0.05) effect of several cultivars on the germination index was discovered (Table 1). The Tanjung cultivar demonstrated the greatest germination index, with an astounding 91.7. The Osaka cultivar came in second with a significant germination index of 84. On the other hand, the Unpad cultivar had a significantly lower germination index (57) (Tables 2 and 3; Fig. 4).

### Lateral roots

**Fig. 5:**
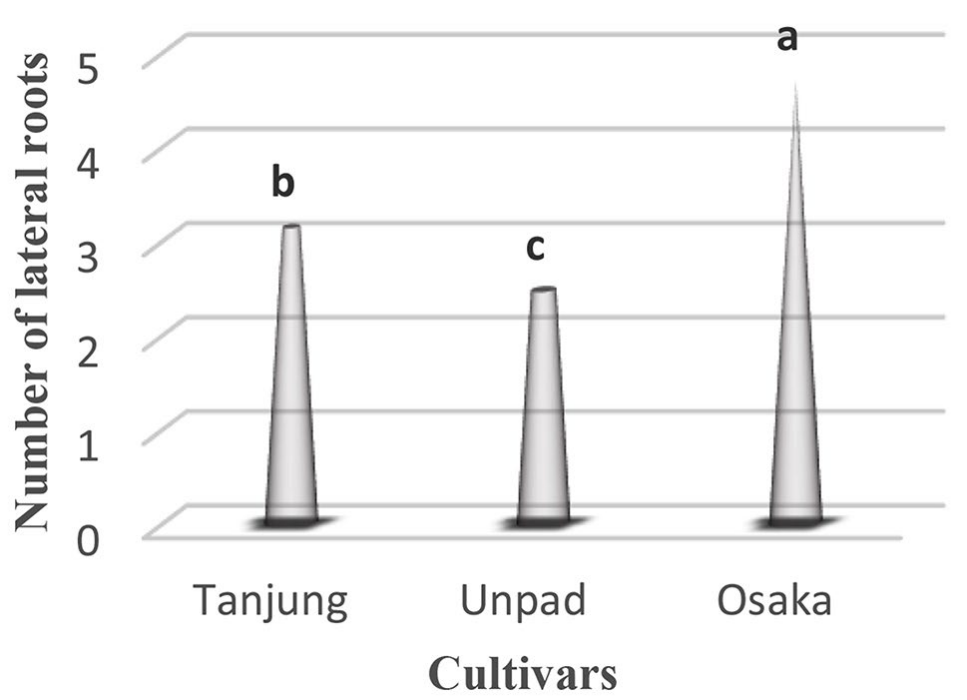
Mean number of lateral roots in cultivars studied under the climatic parameters. (The letters a, b and c indicating the significant differences in cultivars)

The lateral roots showed significant (*p* < 0.05) differences amongst cultivars (Table 1). With an average of 4.7 lateral roots, the Osaka cultivar had the most, indicating strong root development. On the other hand, the Unpad cultivar had the lowest average of 2.47 lateral roots, while the Tanjung cultivar showed a slightly lower standard of 3.14 lateral roots (Tables 2 and 3; Fig. 5).

### Root-shoot length ratio

**Fig. 6:**
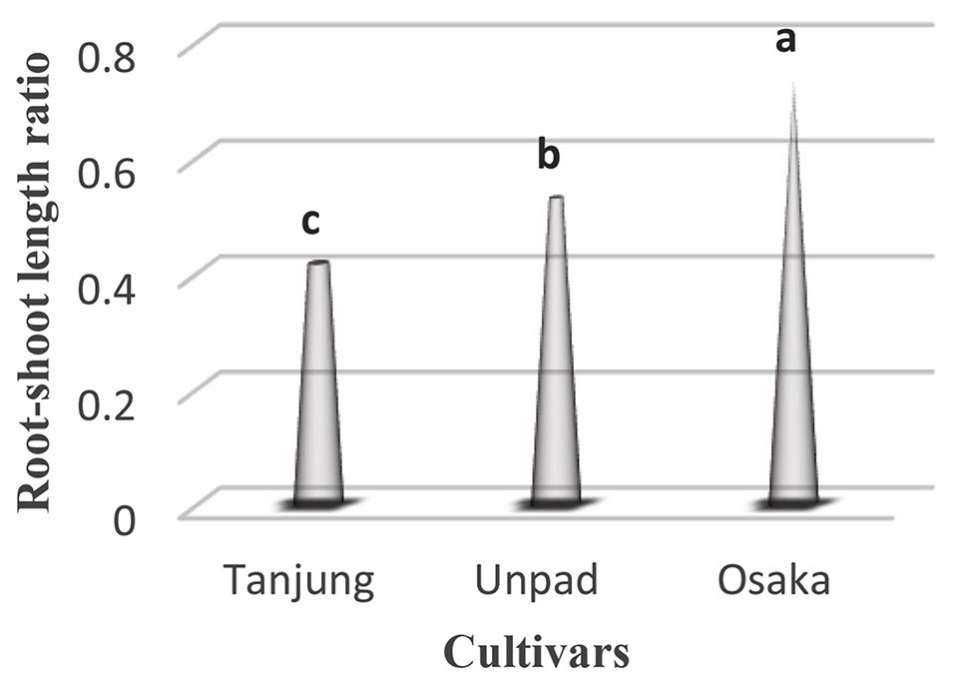
Root-shoot length ratio in cultivars studied under the climatic parameters. (The letters a, b and c indicating the significant differences in cultivars)

Examining the root-shoot length ratio of chili demonstrated significant (*p* < 0.05) patterns across cultivars (Table 1). At an astounding 0.731, the Osaka cultivar demonstrated the most significant root-shoot length ratio. In keeping with this pattern, the Unpad cultivar showed a noteworthy root-shoot length ratio of 0.529. The Tanjung cultivar, on the other hand, displayed a significantly smaller root-shoot length ratio of 0.416 (Tables 2 and 3; Fig. 6).

### Vigor index

**Fig. 7:**
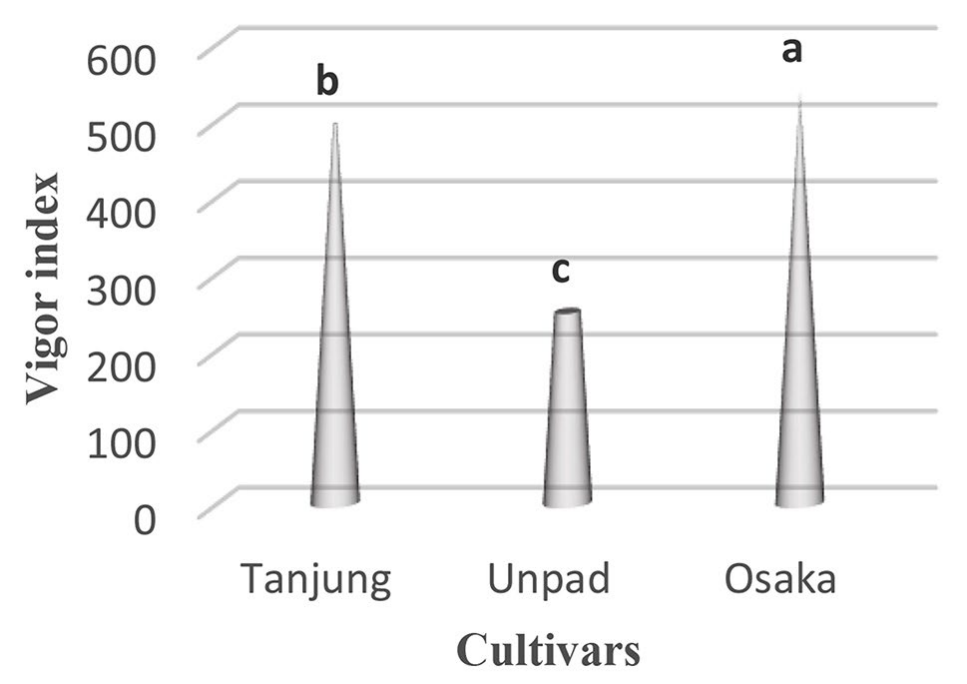
Mean vigor index in cultivars studied under the climatic parameters. (The letters a, b and c indicating the significant differences in cultivars)

The evaluation of the chili vigor index revealed significant (*p* < 0.05) characteristics between cultivars (Table 1). With an outstanding vigor index of 538.4, the Osaka. Closely behind, the Tanjung cultivar showed a high vigor index of 495.04. The Unpad cultivar, on the other hand, had a significantly lower vigor index of 250.2 (Tables 2 and 3; Fig. 7).

### Water absorption capacity (g/seed)

**Fig. 8:**
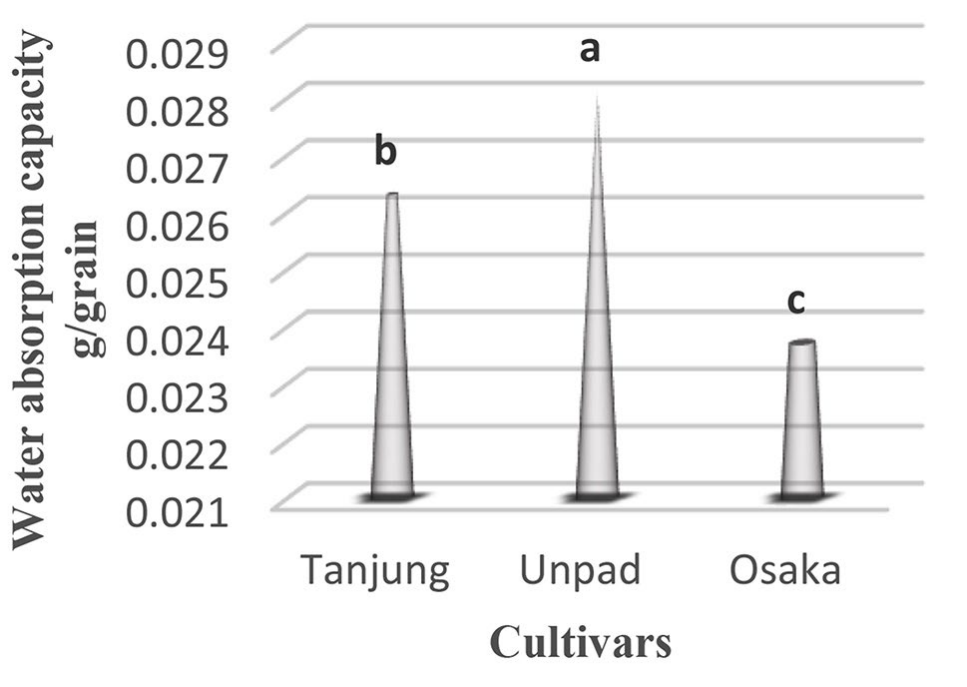
Mean Water absorption capacity in cultivars studied under the climatic parameters. (The letters a, b and c indicating the significant differences in cultivars)

Significant (*p* < 0.05) traits were found among cultivars when the water absorption ability of chili seeds was investigated (Table 1). With a water absorption capacity of 0.028, the Unpad cultivar has the greatest capacity to absorb water. The Tanjung cultivar came in second, with a noteworthy water absorption capacity of 0.026. The Osaka cultivar, on the other hand, had a significantly reduced water absorption capacity of 0.024 (Tables 2 and 3; Fig. 8).

### Water absorption index (%)

**Fig. 9:**
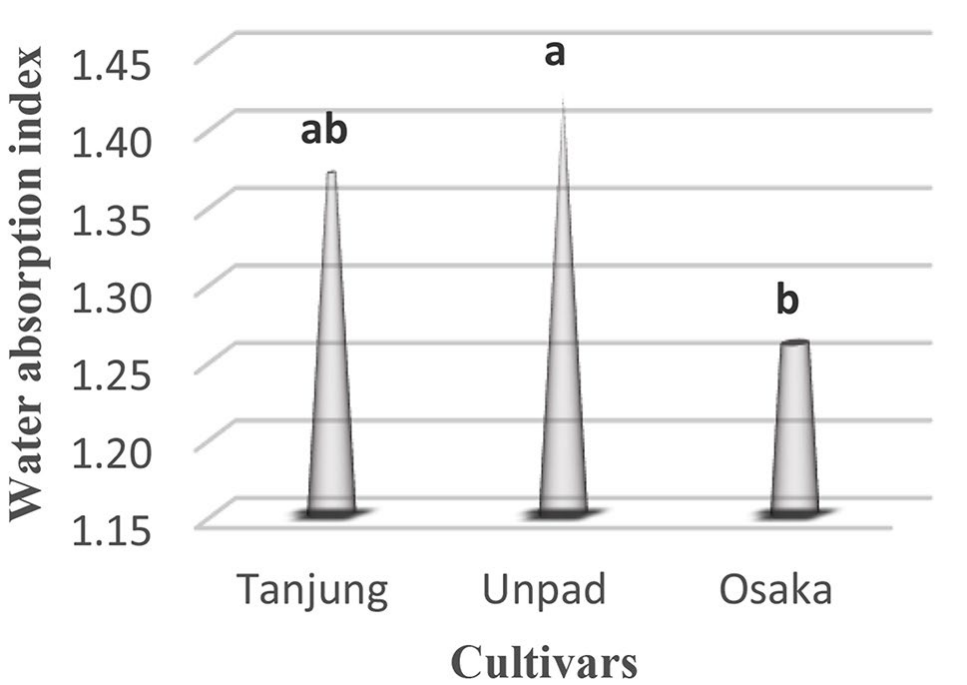
Mean water absorption index in cultivars studied under the climatic parameters. (The letters a, b and c indicating the significant differences in cultivars)

Investigating the water absorption index in chili seeds highlighted significant (*p* < 0.05) traits among cultivars (Table 1). With a 1.42% water absorption index, the Unpad cultivar demonstrated the highest water absorption level. Closely behind, the Tanjung cultivar showed a noteworthy 1.37% water absorption index. The Osaka cultivar, on the other hand, showed a significantly lower water absorption index of 1.26% (Tables 2 and 3; Fig. 9).

### Leaf chlorophyll index

**Fig. 10:**
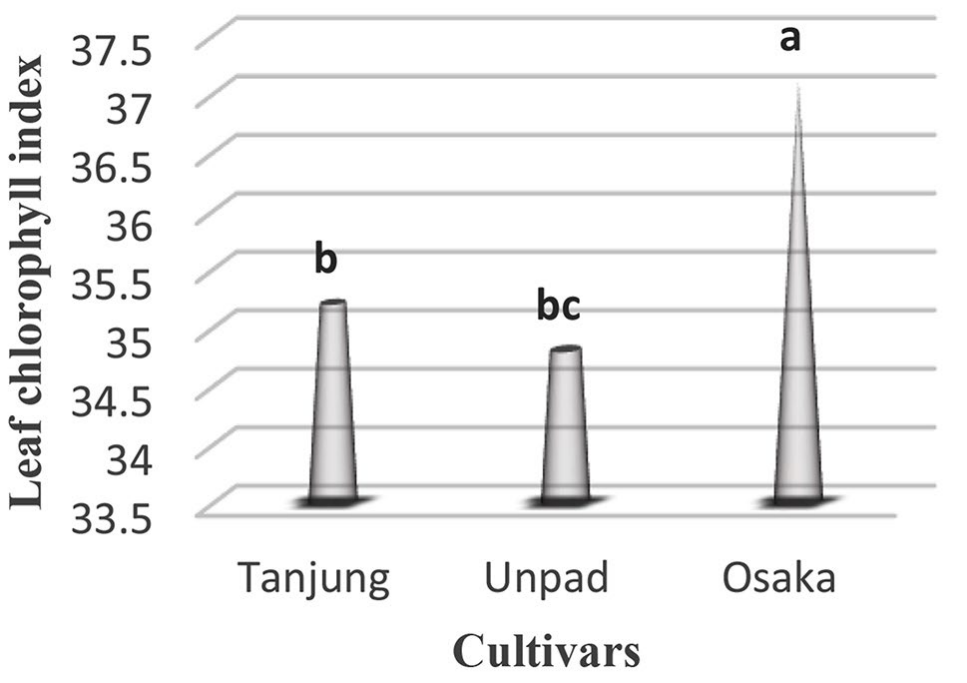
Mean Leaf chlorophyll index in cultivars studied under the climatic parameters. (The letters a, b and c indicating the significant differences in cultivars)

The analysis of the leaf chlorophyll index in chili seeds identified significant (*p* < 0.05) traits for each cultivar (Table 1). With an astounding leaf chlorophyll score of 37.1, the Osaka cultivar had the highest level of chlorophyll concentration. Closely examining the Tanjung cultivar revealed a strong leaf chlorophyll index of 35.2. The Unpad cultivar, on the other hand, had a leaf chlorophyll index of 34.8, which was significantly lower (Tables 2 and 3; Fig. 10).

### Seedling biomass (g)

**Fig. 11:**
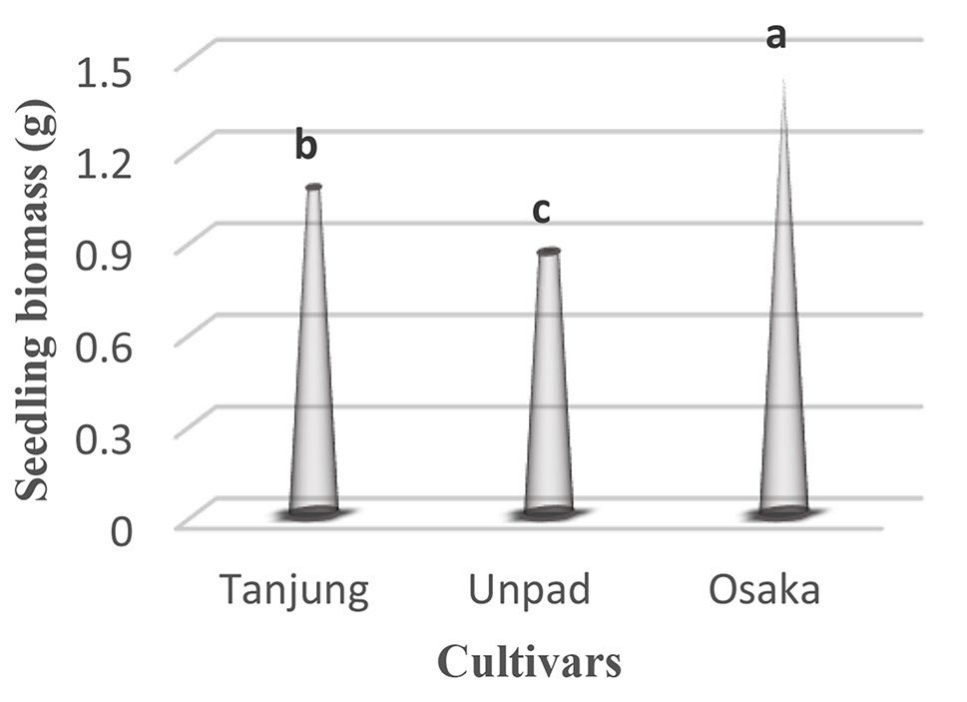
Mean seedling biomass in cultivars studied under the climatic parameters. (The letters a, b and c indicating the significant differences in cultivars)

The study of chili seedling biomass identified significant (*p* < 0.05) patterns between cultivars (Table 1). With a seedling biomass of an excellent 1.429 g, the Osaka cultivar showed the highest level of development, indicating its early solid growth. Closely behind, the Tanjung cultivar showed a noteworthy biomass of 1.068 g in seedlings. The Unpad cultivar, on the other hand, had a significantly lower seedling biomass of 0.857 g (Tables 2 and 3; Fig. 11).

### Irrigation efficiency

**Table 4:**
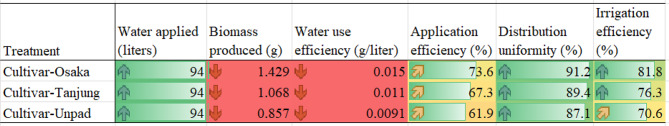
Showing performance of the treatment by heat map in terms of their efficiency towards schedule irrigation practice

In evaluating chili seedling performance under different irrigation conditions, distinct disparities emerged among Osaka, Tanjung, and Unpad cultivars (Table 4). Osaka stood out by producing the highest biomass at 1.429 g, demonstrating superior water use efficiency (WUE) with 0.015 g/liter, and excelling in both application efficiency (73.6%) and distribution uniformity (91.2%). Tanjung followed closely behind Osaka in biomass production (1.068 g), WUE (0.011 g/liter), application efficiency (67.3%), and distribution uniformity (89.4%). On the other hand, Unpad exhibited lower performance across the board, with biomass production at 0.857 g, WUE at 0.0091 g/liter, application efficiency at 61.9%, and distribution uniformity at 87.1%. Overall, Osaka consistently displayed higher irrigation efficiency than Tanjung and Unpad across multiple parameters, emphasizing the significance of cultivar selection in optimizing chili cultivation under varied irrigation conditions.

## Discussion

The germination index is merely one component of a cultivar’s total yield [23]. These differences in the germination indices highlight how crucial it is to comprehend the particular needs of every chili cultivar to ensure successful germination and seedling establishment [24]. Variables like soil conditions, temperature, and moisture content influence germination performances (Fig. 1). It has been discovered that raising the temperature of the root medium impacts pepper plants’ ability to absorb nutrients and develop their root systems [25]. Temperature and precipitation variations in the climate directly affect crop yields, which can affect how effectively chili plants establish their roots [13, 14]. A temperature that is too high might impede the growth of lateral roots (Fig. 1), which may lower plant productivity [15, 26]. Except for the highest air temperature regime (32 °C), the root-shoot ratio tends to decrease as light intensity increases in each air temperature regime [28, 29]. A decrease in the number of roots, shoot length, root length, root-shoot ratio, and fresh weight of seedlings might result from an increase in temperature and exposure time [27, 28]. Varying varieties showed differing responses; generally, cultivars with longer roots are considered to have a higher tolerance to stress [30, 31]. The elevated vigor index indicates a solid and active growth pattern, which may show exceptional genetic qualities enhancing plant vitality [32]. Reflecting the inherent qualities of the cultivars, the observed variations in the vigor index also have consequences for the cultivars’ resilience, prospective yield, and suitability for particular growing environments [33, 34]. The cultivar’s lower vigor index can indicate challenges or restrictions this particular variety may face due to the climate in the area [35]. These results provide novel data about the interactions between environmental and genetic variables and water absorption in chili seeds [36]. Additionally, they offer farmers and seed producers sound advice, assisting them in choosing cultivars based on particular water absorption features [37]. The molecular study might explore the precise genetic processes behind the noted capacities for water absorption, offering a more profound comprehension of these essential characteristics in chili seeds [38]. Comparably, the decreased capacity to absorb water highlights the intricate interaction between genetic and environmental factors that influence seed traits [39]. Genetic variances, seed coat thickness, and internal porosity may all contribute to the variability in water absorption across cultivars [40]. Their internal porosity can influence the water absorption index in seeds [26]. A higher water absorption index cultivar may have improved permeability or structural characteristics that enable effective water uptake [24, 41]. The differences in the permeability and thickness of the seed coat might affect the water absorption indices [42]. The observed differences in leaf chlorophyll index between different cultivars of chilies offer substantial data about how well-adapted each is to the local climate [43]. The cultivar’s impressive chlorophyll concentration indicates a solid capacity to use sunlight for photosynthesis [6]. This elevated chlorophyll content could lead to better energy absorption and usage, providing an advantage over adversaries in the climate [44]. The discrepancy could be explained by genetic changes that affect chlorophyll synthesis or by unfavorable environmental conditions that affect the cultivar [10]. The finding highlights the genetic diversity and flexibility of chili cultivars, since several have proven to have better development traits in particular environmental circumstances [45]. The cultivar’s early robust growth can be ascribed to innate genetic characteristics, such as effective nutrient uptake, improved establishment of roots, and resistance to early-stage growth-related stresses [46, 47]. Genetic variances and environmental elements, including temperature, water availability, and soil composition, may cause these variations in seedling biomass [48].

The findings highlight the significance of cultivar selection in maximizing the cultivation of chilies, as Osaka continuously outperforms Tanjung and Unpad in terms of irrigation efficiency across a range of measures. According to [49], the results support the idea that genetic factors significantly impact plant growth and output. The importance of selecting specific cultivars to improve the efficiency of chili farming under various irrigation circumstances [50]. The observed variations in water use efficiency (WUE) among the cultivars can be attributed to genetic variances in variables, including stomatal conductance, transpiration efficiency, and root design [51]. Cultivars higher WUE indicates that it can use water resources efficiently, which is essential for sustainable agriculture in areas where water is scarce [52]. The superiority of the cultivars’ uniform dispersion and efficient application highlight their versatility with various watering methods [53]. This is consistent with studies highlighting the necessity for agricultural cultivars appropriate for particular environmental factors, such as water availability [54].

## Conclusion

Specifically, the varieties Tanjung, Unpad, and Osaka are the subject of this study, which explores the subtle differences in performance and adaptation between local and imported chili cultivars under various environmental circumstances throughout the germination and early growth stages. Tanjung proves to be an exceptional cultivar, exhibiting strong germination rates and competitive yields. Remarkably, Osaka is just a little behind Tanjung, displaying better qualities such as higher biomass yield, better water use, and even dispersal under different watering schedules. However, Unpad’s poor performance across several criteria raises the possibility that it could be more flexible in response to shifting environmental conditions. These results highlight how important it is to choose cultivars carefully to ensure resilient and sustainable farming techniques for chilies, particularly in the face of erratic climate variations and environmental changes on a worldwide scale. The study supports agricultural diversity and food security by adding to our understanding of cultivar performance under various circumstances, especially in areas like Indonesia, where growing chilies has substantial economic and nutritional benefits.

## Data Availability

Data is provided within the manuscript.
